# Systems Thinking in Public Health: Comment on "Using System Dynamics to Understand Transnational Corporate Power in Diet-Related Non-communicable Disease Prevention Policy-Making: A Case Study of South Africa"

**DOI:** 10.34172/ijhpm.9065

**Published:** 2025-09-30

**Authors:** Martin McKee, Christina Pagel

**Affiliations:** ^1^Department of Health Services Research and Policy, London School of Hygiene & Tropical Medicine, London, UK.; ^2^Clinical Operational Research Unit, University College London, London, UK.

**Keywords:** Systems Analysis, Soft Systems, Complexity, COVID-19

## Abstract

Milson and colleagues illustrate the value of a systems approach to nutrition policy in South Africa. We respond to their call to use systems approaches, and especially, causal loop diagrams (CLDs), more widely in public health. We begin with examples of how systems approaches have provided valuable perspectives on health-related problems and continue with an example of where this approach could have been used but was not, England’s response to the COVID-19 pandemic. We show how an effective response would have required integrated health, economic, and social policies, yet the British government adopted a siloed approach. We conclude by noting that examples of how systems thinking, and specifically CLDs, have been used to support pandemic responses. We conclude by emphasising the need to embed systems thinking in public health policy-making to enhance resilience and preparedness for future crises.

## Introduction

 Milsom and colleagues explore how transnational corporate power impedes the adoption and implementation of measures to prevent diet-related non-communicable diseases in South Africa.^1^ They use qualitative system dynamics to develop causal loop diagrams (CLDs), identifying the feedback processes that entrench this corporate power and create policy inertia while highlighting potential leverage points to counteract this dynamic. In this way, they tear aside the veil that so often conceals this power, which, as they describe, can be instrumental, exerted, for example, through lobbying, structural, whereby the state comes to rely economically on the corporations, or discursive, which involves shaping and, in many cases, controlling the prevailing narrative. They show how the exercise of corporate power leads to policy inertia by emphasising and often exaggerating the complexity of problems and their solutions, failure by the different parts of governments, in ministries and agencies, to come together with a common aim, and the creation of a narrative that creates a false dichotomy between economic growth and health while prioritising the former. Yet they also offer a way forward, highlighting the existence of feedback loops, which can be reinforcing, as when lobbying perpetuates weak health policies, or balancing, as when public discontent or international regulations counter this inertia. They conclude with calls to policy-makers to adopt systems-thinking approaches that can identify strategic interventions that break policy inertia and prioritise healthier environments. In the rest of this commentary, we respond to their call by reflecting on some of the diverse ways in which systems thinking has been used and then focus on a case study where, we argue, it should have been but was not. This is the response to the COVID-19 pandemic in England.

## Systems Thinking in Public Health

 Systems thinking is especially useful when the systems involved are complex and adaptive. Such systems have several characteristics. The first is path dependency, whereby starting conditions matter. For example, power hierarchies created at the outset can be very difficult to change, although they are susceptible to shocks. Second, they have multiple feedback loops, both reinforcing and balancing as described above. Third, the interactions between elements are often non-linear. More of one may have a large or small, or even no effect, depending on circumstances. Fourth, each system comprises multiple sub-systems that interact with one another, each involving a Transformation, such as the production of something, undertaken by Actors, for the benefit of Customers, in ways that are aligned with a Weltanschauung, or vision of the world, subject to Owners, who are able to stop the system from operating, all subject to Environmental constraints (often referred to by the mnemonic CATWOE).^2^ Examples from our work include, for example, the systems required to ensure effective care for patients with type 1 diabetes,^3^ which involve sub-systems for procuring and distributing medicines and diagnostics, for training health workers and empowering patients, and for developing guidelines and referral mechanisms. Thus, programmes to train health workers depend on school systems that give them essential basic skills and distribution systems that mean that there is insulin in their pharmacy. Cancer screening depends on other parts of government creating and maintaining population registers that can identify eligible individuals and cancer registries that track outcomes and feedback information on problems.^4^ A plan to increase medical school numbers in the United Kingdom failed to consider labour shortages that limited the ability to build new lecture theatres and the ability of health facilities to offer sufficient clinical placements.^5^

 Milson and colleagues illustrate the relationships in their analysis with a CLD.^1^ The value of this approach can also be seen in some other examples. Hosseinichimeh and colleagues have developed a CLD for understanding why efforts to reduce youth drink-driving have stalled in the United States over the last 20 years.^6^ They identify several reinforcing loops, including the impacts of marketing and advertising and of peers and parents, as well as balancing loops such as traffic stops and near misses. This allowed them to identify policy levers, such as discouraging teens from binge drinking or changing perceptions of the likelihood of traffic stops.

 Another example is the prevention of sexually transmitted diseases. Chea and colleagues used CLDs to explore the context and wider determinants of sexual risk-taking behaviours in young people in Kenya.^7^ They identified several reinforcing loops, ranging from sex tourism to gender-based violence to alcohol/drug use to misinformation to family environment. Their systems thinking approach highlighted the importance of immediate means of preventing risk taking behaviour, such as promoting condom use, but also distal levers such as reducing poverty by increasing state support for university students.

 As these examples illustrate, the potential for systems thinking methods more broadly, and CLDs in particular, to support more effective policy-making across a whole range of public health domains is enormous. As Milsom and colleagues^1^ and Hosseinichimeh and colleagues both show elegantly, the latter in the alcohol sector,^6^ it is increasingly important to include the behaviours of commercial actors. However, the applications are much wider and, in the next section, we highlight the importance of systems thinking in emergencies.

## From Systems Thinking for the Everyday to Pandemics

 The preceding examples illustrate the broad range of health-related topics amenable to qualitative systems analysis and the use of CLD. In the remainder of this commentary, we focus on their potential in a public health emergency and, specifically, a pandemic.

 There were several examples from the COVID-19 pandemic. For example, Burke and colleagues used them to understand the interplay between protective behaviours (such as mask-wearing), self-isolation, and travel in the United States^8^ and Hsiao et al developed CLDs to understand Taiwan’s successful pandemic approach.^9^

 Sahin et al developed a comprehensive general CLD that, while designed to understand the complexity of the pandemic, has much wider applicability.^10^ Their diagram is colour-coded: Green for economic, blue for social, purple for health, and orange for environmental factors. It shows a range of reinforcing and balancing feedback loops. For example, economic activities affect gross domestic product, unemployment, and business closures, which in turn impact supply chains and mental well-being. Social factors, including misinformation and panic, influence trust in governments and crime levels. Health-related elements, such as numbers of confirmed cases and healthcare capacity, affect vulnerable populations and hygiene practices. Environmental factors, including waste generation, are linked to economic and health factors. Government interventions, such as restrictions, stimulus packages, and awareness campaigns, interact with these domains to either mitigate or exacerbate problems. Sahin and colleagues’ CLD highlights beautifully the reinforcing loops within the health system, population, interventions and economy and how they interact with each other.

 Such approaches did, however, find only limited use. We now consider how they could have helped in one country, England, that prior to the pandemic was assessed as having a very high degree of preparedness but which, at least until the vaccine became available, performed poorly. We explore how systems analysis could have informed more effective responses to the COVID-19 pandemic in England by identifying key feedback loops that needed to be addressed when considering pandemic control measures but often were not.

 Shortly after the onset of the pandemic, a paper co-authored by one of us explored the potential consequences of the immediate responses that were being implemented.^11^ There was an urgent need to interrupt the transmission of a highly contagious virus that spread through the air, with cases increasing exponentially. Even a few days’ delay was catastrophic.^12^ However, necessary measures, such as restrictions on mixing and closures of schools and workplaces, would have consequences.

 These consequences included economic downturns, leading to widespread unemployment and income insecurity that, in turn, have cascading effects, including worsening mental health and deepening health inequalities. Particularly vulnerable groups, such as individuals in precarious jobs or those without social safety nets, would be disproportionately affected. In such a situation, policy responses should be progressive and integrated, combining health, economic, and social measures to create a comprehensive response. This would require a multiagency response, bringing together expertise from various sectors and disciplines to address the diverse challenges posed by the pandemic. Collaboration and integration across systems would be essential for effective mitigation and recovery.

 In reality, this rarely happened. Too often, responses were siloed. Organisations, including departments and other bodies within government, worked in isolation with little understanding of what others could contribute in terms of intelligence, knowledge, or resources. For example, a vastly expensive test and trace system ignored local public health teams with long experience of contract tracing and local knowledge.^13^ A “VIP” lane for purchasing personal protective equipment operated outside the existing procurement system, leading to massive waste.^14^ Coordination between hospitals and social care facilities was almost non-existent.^15^

 Yet, systems analysis could have mapped the interconnections and feedback loops that defined the crisis. The pandemic’s spread and its impacts were not linear; they were shaped by complex dynamics, such as the strain on healthcare systems caused by rising hospitalisations, which in turn led to staff burnout that reduced capacity to manage subsequent waves. System dynamics modelling could have captured these relationships, enabling governments to anticipate breaking points and design interventions to prevent system collapse.

 Systems analysis could also have illuminated the difficult trade-offs faced by policy-makers. Lockdowns, for instance, slowed the virus’s spread but disrupted economies and exacerbated mental health challenges. Using models to simulate these scenarios would have helped decision-makers understand the long-term ripple effects of their actions. The policy of closing schools during the pandemic exemplifies this. What would be needed to maintain education while interrupting virus transmission?

 There are many ways in which a similar map might have helped avoid some of the negative reinforcing loops that the UK experienced. Thus, the pandemic disproportionately affected marginalised communities, exposing underlying health and social disparities. By examining how social determinants of health, such as overcrowded housing, poor access to healthcare, and precarious employment, interacted with the virus, systems analysis could have targeted interventions where they were most needed. Globally, it could have highlighted the consequences of vaccine hoarding by wealthy nations, illustrating how inequitable distribution delayed global pandemic control.

 One of the pandemic’s defining challenges was the rapid spread of disinformation, which was widespread in the debate on how schools should respond to the pandemic. Systems analysis could have explored how disinformation proliferated across social media, creating self-reinforcing cycles of doubt and mistrust. By identifying leverage points, such as the role of trusted community leaders in countering false narratives, systems thinking could have informed strategies to combat disinformation effectively. Additionally, models could have examined how public perception of government responses influenced compliance, shaping the trajectory of the pandemic.

 Such a systems approach would also be helpful in tackling the long-term consequences of the pandemic. The cascading effects of COVID-19 on mental health, education, and global supply chains highlighted how interconnected systems respond to crises. For instance, many individuals faced heightened isolation, economic uncertainty, and disrupted social networks during lockdowns. Improved understanding of feedback loops linking these factors to mental health could have helped design interventions targeting those most vulnerable. Similarly, disruptions in supply chains due to travel restrictions and changing consumer behaviour could have been modelled to identify bottlenecks and improve resilience, as was done elsewhere.

## Conclusion

 There is an urgent need for more systems thinking in public health, both in everyday life and for emergency response. Governments and organisations that embraced systems approaches, like New Zealand, which tightly integrated border controls, testing, and contact tracing, demonstrated effective pandemic management. Systems analysis provided a framework for understanding how policies influenced the virus’s spread and helped minimise unintended consequences.

 Pandemics are not merely a public health emergency; they represent a crisis of interconnected systems. Systems analysis offers the tools to navigate such complexity by providing insights into the relationships between social, economic, and health factors. Systems thinking enables policy-makers to anticipate challenges, design equitable solutions, and build resilience by simulating interventions, mapping feedback loops, and highlighting vulnerabilities. As the world prepares for future crises, systems analysis must become a cornerstone of public health practice, transforming how we understand and respond to global challenges.

## Ethical issues

 Not applicable.

## Conflicts of interest

 Authors declare that they have no conflicts of interest.
